# Supplementary material for: Automatically disambiguating medical acronyms with ontology-aware deep learning
Source: Nat Commun. 2021 Sep 7;12:5319. doi: 10.1038/s41467-021-25578-4 (PMC8423722; doi:10.1038/s41467-021-25578-4)
Supplement: Supplementary file 1 — Supplementary Information [file 41467_2021_25578_MOESM1_ESM.pdf]

Supplementary Information

Supplementary Figure 1. **Website we developed containing a sample sentence from i2b2 that we presented to hand labellers.** (a) The abbreviation was highlighted and bolded in the sentence, and words within a context window of 5 were bolded for easier viewing. (b, c) The users were asked to select the expansion from AllAcronyms that best suited the sentence, which was presented in a drop-down menu. If none of the expansions made sense, there was an option to select “None” or “Ambiguous”. We removed these samples from the test set.

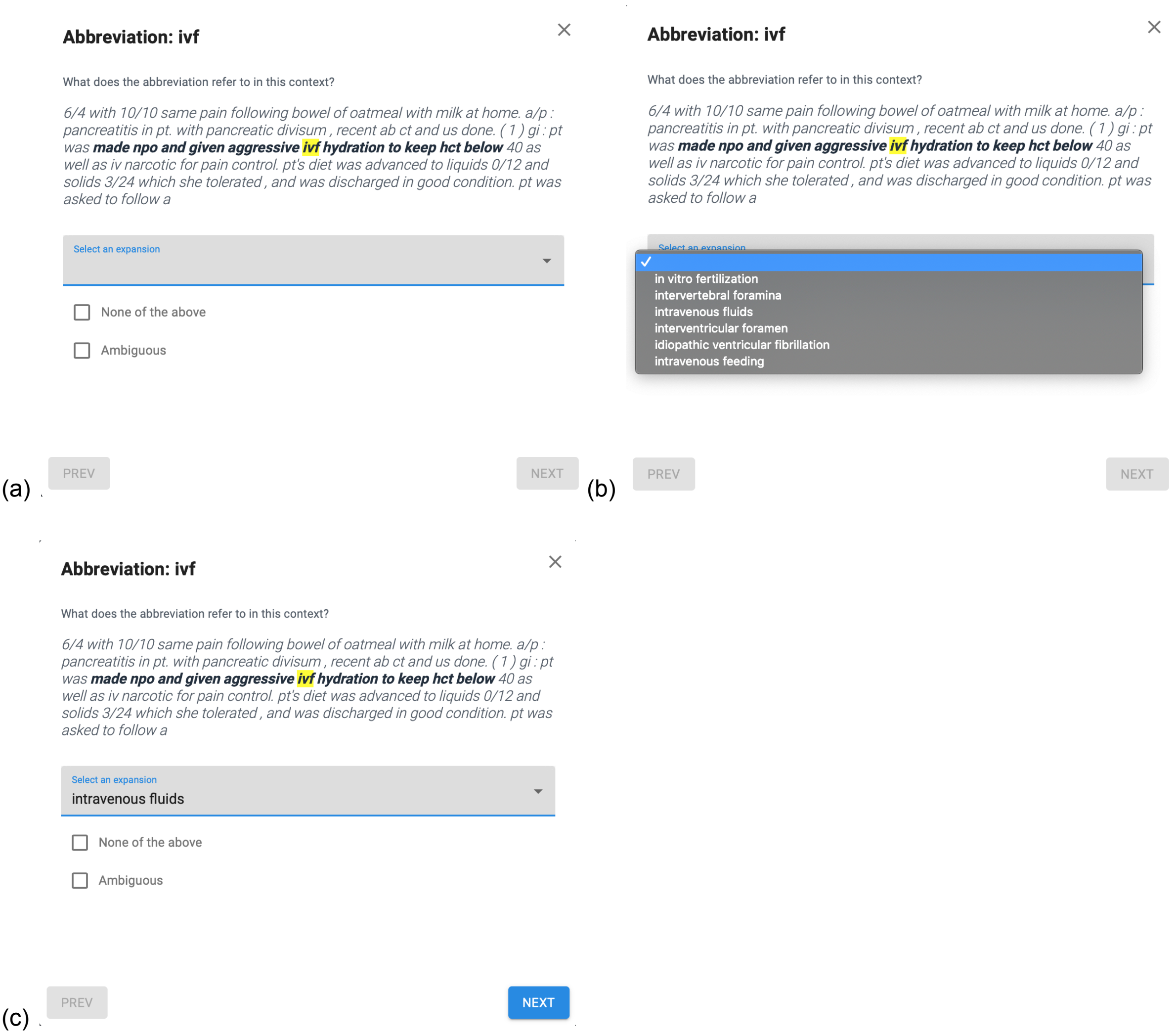

Supplementary Table 1. **List of abbreviations from i2b2 we hand-labelled for an auxiliary test set.** We show the abbreviations we considered, the list of possible expansions, the number of sentences containing each expansion. We removed any sentences that had not enough context for disambiguation, spelling mistakes, unrelated concepts, or proper nouns (marked with "OTHER"), as well as expansions that were abbreviations themselves. After applying this filter, 24 abbreviations had more than one expansion. We used these in the final test set.

Abbreviation to be considered	Expansion:Number sentences labelled with expansion
afb	acid fast bacillus:7/aflatoxin b:1
ain	acute tubulo interstitial nephritis:2/anal intraepithelial neoplasia:1/(OTHER:3)
avn	atrioventricular node:1/avascular necrosis of bone:3
ccu	critical care unit:28/coronary care unit:21
cva	cerebral vascular accidents:43/costovertbral angle:7
cvp	central venous pressures:15/cyclophosphamide, vincristine, prednisone:2
ddi	didanosine:2/dual-chamber pacing, dual-chamber sensing, inhibited response:5
derm	dermatological:15/dermatologist:7
disp	dispense:35/disposition:5
dtr	deep tendon reflexes:4/due to recent:1/daughter:1
gms	general medical services:20/grams:2
hsm	hepatosplenomegaly:29/holosystolic murmur:12
ivf	in vitro fertilization:2/intravenous feeding:2/intravenous fluids:45/(OTHER:1)
loc	loss of consciousness:15||laxative of choice:1
mvr	mitral valve repair:1/mitral valve replacements:16/(OTHER:13)
osm	osmolarity:3/osmotic:1
ou	oculus uterque:46/observation unit:1/(OTHER:1)
pacs	picture archiving and communication systems:1/premature atrial contractions:4
phos	phosphatase:30/phosphate:20
pvd	peripheral vascular diseases:37/pulmonary valve disease:9/posterior vitreous detachment:1
sbp	spontaneous bacterial peritonitis:4/systolic blood pressures:46
sma	smooth muscle:18/superior mesenteric arteries:7
vac	vacuum assisted closure:20/vaccine:2
xl	expression levels:1/extended release:49

Supplementary Dataset 1. **Full list of 270 abbreviations presented to medical students for hand labelling and location of sentences in i2b2.** We show the abbreviations we considered, the list of possible expansions, and the number of sentences containing each expansion. We marked any expansions that we did not use with round brackets: these were located in sentences that did not have enough context for disambiguation, spelling mistakes, unrelated concepts, or proper nouns (marked with "OTHER"), as well as expansions that were abbreviations themselves. In the third column, we indicate where we found the abbreviations within the i2b2 dataset. The column is formatted as follows:

{expansion_1}:::{note_1_name}:{starting_index_of_abbreviation}|{note_2_name}:{starting_index_of_abbreviation} ///{expansion_2}:::{note_1_name}:{starting_index_of_abbreviation. Note that we removed the filetype from the note name (i.e. ".txt", ".xml"), and we replaced all whitespace characters with a single space (i.e. `note = re.sub(r"\s\s+", " ", note).lower()`, where `re` is the Python regular expression library).

This dataset is located in a separate spreadsheet.
