# Supplementary material for: Automatically disambiguating medical acronyms with ontology-aware deep learning
Source: Nat Commun. 2021 Sep 7;12:5319. doi: 10.1038/s41467-021-25578-4 (PMC8423722; doi:10.1038/s41467-021-25578-4)
Supplement: Supplementary file 3 — Description of Additional Supplementary Files [file 41467_2021_25578_MOESM3_ESM.pdf]

Title: Supplementary Dataset 1.

Description: Full list of 270 abbreviations presented to medical students for hand labelling and location of sentences in i2b2. We show the abbreviations we considered, the list of possible expansions, and the number of sentences containing each expansion. We marked any expansions that we did not use with round brackets: these were located in sentences that did not have enough context for disambiguation, spelling mistakes, unrelated concepts, or proper nouns (marked with "OTHER"), as well as expansions that were abbreviations themselves. In the third column, we indicate where we found the abbreviations within the i2b2 dataset. The column is formatted as follows:

{expansion_1}:::{note_1_name}:{starting_index_of_abbreviation}|{note_2_name}:{starting_index_of_abbreviation} ///{expansion_2}:::{note_1_name}:{starting_index_of_abbreviation. Note that we removed the filetype from the note name (i.e. ".txt", ".xml"), and we replaced all whitespace characters with a single space (i.e. `note = re.sub(r"\s\s+", " ", note).lower()`, where `re` is the Python regular expression library).
