# Supplementary material for: Automatically disambiguating medical acronyms with ontology-aware deep learning
Source: Nat Commun. 2021 Sep 7;12:5319. doi: 10.1038/s41467-021-25578-4 (PMC8423722; doi:10.1038/s41467-021-25578-4)
Supplement: Supplementary file 4 — Reporting Summary [file 41467_2021_25578_MOESM4_ESM.pdf]

nature research

Corresponding author(s): Marta Skreta; Michael Brudno

Last updated by author(s): Jul 20, 2021

Reporting Summary

Nature Research wishes to improve the reproducibility of the work that we publish. This form provides structure for consistency and transparency in reporting. For further information on Nature Research policies, see our Editorial Policies and the Editorial Policy Checklist.

Statistics

For all statistical analyses, confirm that the following items are present in the figure legend, table legend, main text, or Methods section.

n/a	Confirmed	
☐	☒	The exact sample size (*n*) for each experimental group/condition, given as a discrete number and unit of measurement
☐	☒	A statement on whether measurements were taken from distinct samples or whether the same sample was measured repeatedly
☐	☒	The statistical test(s) used AND whether they are one- or two-sided<br>*Only common tests should be described solely by name; describe more complex techniques in the Methods section.*
☐	☒	A description of all covariates tested
☐	☒	A description of any assumptions or corrections, such as tests of normality and adjustment for multiple comparisons
☒	☐	A full description of the statistical parameters including central tendency (e.g. means) or other basic estimates (e.g. regression coefficient) AND variation (e.g. standard deviation) or associated estimates of uncertainty (e.g. confidence intervals)
☐	☒	For null hypothesis testing, the test statistic (e.g. *F*, *t*, *r*) with confidence intervals, effect sizes, degrees of freedom and *P* value noted<br>*Give P values as exact values whenever suitable.*
☒	☐	For Bayesian analysis, information on the choice of priors and Markov chain Monte Carlo settings
☐	☒	For hierarchical and complex designs, identification of the appropriate level for tests and full reporting of outcomes
☒	☐	Estimates of effect sizes (e.g. Cohen's *d*, Pearson's *r*), indicating how they were calculated

Our web collection on statistics for biologists contains articles on many of the points above.

Software and code

Policy information about availability of computer code

Data collection	Data was collected from publicly available sources (CASI, MIMIC, i2b2, AllAcronyms). Preprocessing scripts (to standardize punctuation and alphanumeric characters) and scripts for reverse substitution are available in a public GitHub repository (https://github.com/martaskrt/abbr_disamb). The following software was used during model training:<br>Python3 (3.7)<br>Tensorflow (1.13.1)<br>fasttext (0.9.2)<br>sklearn (0.20.0)<br>pandas (0.24.2)<br>hyperopt (0.2.3)<br>Pytorch (1.5.0)<br>transformers (4.0.1)
Data analysis	To analyze data, standard online statistical packages were used. Specifically, scipy.stats.wilcoxon (version 1.4.1) was used to obtain p-values where applicable. Scripts for computing micro and macro accuracies are available in a public GitHub repository (https://github.com/martaskrt/abbr_disamb).

For manuscripts utilizing custom algorithms or software that are central to the research but not yet described in published literature, software must be made available to editors and reviewers. We strongly encourage code deposition in a community repository (e.g. GitHub). See the Nature Research guidelines for submitting code & software for further information.

Data

Policy information about availability of data

All manuscripts must include a data availability statement. This statement should provide the following information, where applicable:

- Accession codes, unique identifiers, or web links for publicly available datasets
- A list of figures that have associated raw data
- A description of any restrictions on data availability

The following databases were used in this study:

MIMIC-III (https://physionet.org/content/mimiciii/1.4/, version 1.4)
CASI (https://conservancy.umn.edu/handle/11299/137703)
i2b2 (https://www.i2b2.org/NLP/DataSets/Main.php)
UMLS (https://uts.nlm.nih.gov/uts/umls/home)
AllAcronyms (https://www.allacronyms.com/_medical)

We have published our code on how to generate training/validation/test sets from these datasets. Note that to make use of our code base, one must first obtain access to MIMIC-III, i2b2, and/or UMLS. For the i2b2 hand labelled dataset, we have noted the location of each abbreviation and its expansion in Supplementary Table S2. We are unable to provide clinical notes from The Hospital for Sick Children due to privacy restrictions.

Field-specific reporting

Please select the one below that is the best fit for your research. If you are not sure, read the appropriate sections before making your selection.

☒ Life sciences ☐ Behavioural & social sciences ☐ Ecological, evolutionary & environmental sciences

For a reference copy of the document with all sections, see nature.com/documents/nr-reporting-summary-flat.pdf

Life sciences study design

All studies must disclose on these points even when the disclosure is negative.

Sample size	Sample size = 1205 abbreviations with approx. 600 training samples, 200 validation samples, and 200 test samples per abbreviation. Number of samples chosen because that was only available data to train/test on.
Data exclusions	No data was excluded.
Replication	Findings are reproducible; code run at least three times independently to ensure findings were the same.
Randomization	Samples were randomly sorted before splitting into train/validation/test sets where applicable.
Blinding	Investigators had no insight into the randomization algorithms used to split the data and so could be considered blinded.

Reporting for specific materials, systems and methods

We require information from authors about some types of materials, experimental systems and methods used in many studies. Here, indicate whether each material, system or method listed is relevant to your study. If you are not sure if a list item applies to your research, read the appropriate section before selecting a response.

Materials & experimental systems

n/a	Involved in the study	
☒	☐	Antibodies
☒	☐	Eukaryotic cell lines
☒	☐	Palaeontology and archaeology
☒	☐	Animals and other organisms
☒	☐	Human research participants
☒	☐	Clinical data
☒	☐	Dual use research of concern

Methods

n/a	Involved in the study	
☒	☐	ChIP-seq
☒	☐	Flow cytometry
☒	☐	MRI-based neuroimaging
